# Inhaled tolafentrine reverses pulmonary vascular remodeling via inhibition of smooth muscle cell migration

**DOI:** 10.1186/1465-9921-6-128

**Published:** 2005-11-01

**Authors:** Soni Pullamsetti, Stefanie Krick, Hüseyin Yilmaz, Hossein Ardeschir Ghofrani, Christian Schudt, Norbert Weissmann, Beate Fuchs, Werner Seeger, Friedrich Grimminger, Ralph Theo Schermuly

**Affiliations:** 1 University of Giessen Lung Center (UGLC), Medical Clinic II/V, Giessen, Germany; 2 Altana Pharma, Constance, Germany

## Abstract

**Background:**

The aim of the study was to assess the chronic effects of combined phosphodiesterase 3/4 inhibitor tolafentrine, administered by inhalation, during monocrotaline-induced pulmonary arterial hypertension (PAH) in rats.

**Methods:**

CD rats were given a single subcutaneous injection of monocrotaline to induce PAH. Four weeks after, rats were subjected to inhalation of tolafentrine or sham nebulization in an unrestrained, whole body aerosol exposure system. In these animals (i) the acute pulmonary vasodilatory efficacy of inhaled tolafentrine (ii) the anti-remodeling effect of long-term inhalation of tolafentrine (iii) the effects of tolafentrine on the expression profile of 96 genes encoding cell adhesion and extracellular matrix regulation were examined. In addition, the inhibitory effect of tolafentrine on ex vivo isolated pulmonary artery SMC cell migration was also investigated.

**Results:**

Monocrotaline injection provoked severe PAH (right ventricular systolic pressure increased from 25.9 ± 4.0 to 68.9 ± 3.2 after 4 weeks and 74.9 ± 5.1 mmHg after 6 weeks), cardiac output depression and right heart hypertrophy. The media thickness of the pulmonary arteries and the proportion of muscularization of small precapillary resistance vessels increased dramatically, and the migratory response of ex-vivo isolated pulmonary artery smooth muscle cells (PASMC) was increased. Micro-arrays and subsequent confirmation with real time PCR demonstrated upregulation of several extracellular matrix regulation and adhesion genes, such as matrixmetalloproteases (MMP) 2, 8, 9, 10, 11, 12, 20, Icam, Itgax, Plat and serpinb2. When chronically nebulized from day 28 to 42 (12 daily aerosol maneuvers), after full establishment of severe pulmonary hypertension, tolafentrine reversed about 60% of all hemodynamic abnormalities, right heart hypertrophy and monocrotaline-induced structural lung vascular changes, including the proportion of pulmonary artery muscularization. The upregulation of extracellular matrix regulation and adhesion genes was reduced by nearly 80% by inhalation of the tolafentrine. When assessed in vitro, tolafentrine blocked the enhanced PASMC migratory response.

**Conclusion:**

In conclusion, we demonstrate for the first time that inhalation of combined PDE3/4 inhibitor reverses pulmonary hypertension fully developed in response to monocrotaline in rats. This "reverse-remodeling" effect includes structural changes in the lung vascular wall and key molecular pathways of matrix regulation, concomitant with 60% normalization of hemodynamics.

## Background

Pulmonary arterial hypertension (PAH) is a severe disabling disease characterized by elevation of pulmonary artery pressure and death attributable to right heart failure [1]. It is a progressive, proliferative vascular disorder resulting from persistent vasoconstriction and structural remodeling of pulmonary vessels. The structural changes include endothelial cell injury, neovascularization of small arteries, smooth muscle cell (SMC) migration and proliferation, and abnormal accumulation of extracellular matrix proteins associated with activation of matrix metalloproteinases (MMPs) [2].

The MMPs are a family of matrix-degrading enzymes that have been implicated in two important processes in vessel wall repair: cellular migration [3], and regulating extracellular matrix composition and content [4]. Based on their substrate specificity, MMPs were subdivided into four groups: i) interstitial collagenases (MMP 1, MMP 8 and MMP 13) that degrade fibrillary collagens; ii) type IV collagenases (MMP 2 and MMP 9) that degrade basement membrane components; iii) stromelysins (MMP 3, MMP 10 and MMP 11) that degrade proteoglycans, fibronectin, laminin, gelatin and the globular proteins of the type IV collagen; and iv) membrane type-MMPs (MT-1, MMP 15, MMP 16 and MMP 17), possessing a broad spectrum of activities.

Of the MMPs, expression of MMP 2 and MMP 9 in particular, which degrade type IV collagen of basement membranes, are increased in the pulmonary vascular bed, during both monocrotaline (MCT) and hypoxia-induced experimental PAH [5]. In addition, MMP 1, an interstitial collagenase, is upregulated in MCT-induced PAH [6]. In recent years, several pharmacological interventions in PAH, including MMP inhibitors, endothelin antagonist, angiotensinogen inhibitors and phosphodiesterase (PDE) inhibitors that target either the MMP cascades or endogenous vascular elastases, proved to be beneficial in experimental PAH models, with most of these agents being applied prior to full establishment of the disease [7-11]. Suppression of vessel wall remodeling was assumed to largely contribute to these beneficial effects.

The phosphodiesterases (PDEs) are a large family of intracellular enzymes that degrade cyclic nucleotides [12,13]. Because of their potential for altering a variety of cellular responses, PDEs are appealing targets for the treatment of PAH [14,15]. Phosphodiesterase 3 and 4 isoenzymes are the essential players co-regulating cAMP catabolism in many organs, including the lung, and were shown to be upregulated in experimental PAH models. Inhibitors of PDE 3 and 4 synergistically promoted the acute pulmonary vasodilation evoked by prostacyclin or its stable analogues in experimental models of PAH [16-18]. Recently, inhalation of the combined -selective PDE3/4 inhibitor tolafentrine has been shown to amplify the vasodilatory effect of inhaled iloprost in patients with PAH [18]. However, no data are currently available regarding effects of long-term inhalation of tolafentrine on hemodynamics and pulmonary vascular remodeling in PAH models.

In the present investigation, we employed monocrotaline (MCT), a toxin derived from plants of the *Crotalaria *species [19], for pulmonary artery smooth muscle cell hypertrophy and severe pulmonary hypertension in rats [20]. In this model we examined (i) the acute pulmonary vasodilatory efficacy of inhaled tolafentrine (ii) the anti-remodeling effect of long-term inhalation of tolafentrine (iii) the inhibitory effect of tolafentrine on pulmonary artery SMC cell migration, and (iv) the effect of the PDE inhibitor on the expression profile of 96 genes encoding cell adhesion and extracellular matrix regulation. To mimic clinical conditions, inhalation of tolafentrine commenced after pulmonary hypertension was already fully established. Essentially, we found that inhaled tolafentrine reverses hemodynamic abnormalities as well as structural changes, SMC migration and proliferation and matrix remodeling, representing the key features of MCT induced PAH.

## Methods

### Animal experiments

Experiments were performed on male CD rats 300 – 350 g body weight, Charles River, Sulzfeld, Germany). Pulmonary hypertension was induced by a single subcutaneous injection of monocrotaline (MCT, 60 mg/kg, Sigma, Deishofen, Germany), dissolved in 0.1 M NaOH, adjusted to pH 7.4 with 0.1 M HCl, according to the previous reports [7,10,21,22].

The experiments were performed in accordance with the National Institutes of Health Guidelines on the Use of Laboratory Animals. Both the University Animal Care Committee and the Federal Authorities for Animal Research of the Regierungspräsidium Giessen (Hessen, Germany) approved the study protocol.

### Study groups

The animals were classified into the following four groups: 1) rats injected with saline sacrificed after 28 days (Control, n = 8); 2) MCT-injected rats sacrificed after 28 days (MCT_[28d]_, n = 12); 3) MCT-injected sacrificed after 42 days, with nebulized vehicle being administered from day 28 to day 42 (MCT_[42d]_, n = 14); 4) MCT-injected rats sacrificed after 42 days, with nebulized tolafentrine being administered from day 28 to day 42 (MCT_[42d]_/Tola, n = 10). For acute hemodynamic studies 12 MCT_[28d] _rats (MCT injected for 28 days) were used (inhalation of saline, n = 6; and inhalation of tolafentrine in two different doses, n = 6 each).

### Inhalation of tolafentrine

Four weeks after a single MCT injection, rats were subjected to inhalation of tolafentrine or sham nebulization in an unrestrained, whole body aerosol exposure system as described [23]. For assessment of chronic effects of inhaled saline or tolafentrine (dose deposited in the lungs ~ 120 μg/kg day), 15 min nebulization maneuvers using a jet nebulizer with a constant flow rate of 6 l/min (Pari LC Star, Pari, Starnberg, Germany) were repeated twelve times per day for 2 weeks (day 28 – 42). Particles generated by the jet nebulizer were characterized by a mass median aerodynamic diameter (MMAD) of 2.8 μm and a geometric standard deviation (GSD) of 2.5 (determined by laser difractometric measurements, as described [24]. Preliminary experiments with nebulization of ^99^Tc-DTPA determined the total lung deposition of nebulized material to range at 0.5 %, in accordance with previous studies in this model [22].

To assess the acute effects of inhaled tolafentrine, hemodynamic testing in response to the drug was performed in animals that had previously received a MCT injection 4 weeks prior to study. Hemodynamics were measured before and after a single inhalation of the tolafentrine (130 and 650 μg/kg min for 10 min) by an ultrasonic nebulizer with MMAD of 4.0 μm and GSD of 2.1 as described previously [22,23,25].

### Surgical Preparation, measurement of hemodynamics and tissue preparation

For measurement of hemodynamic parameters, rats were anaesthetized with an i.p. injection of ketamine (9 mg/kg body mass) and medetomidine (100 μg/kg body mass), followed by an i.m. injection of atropine (250 μg/kg body mass). The rats were tracheotomized and ventilated with a frequency of 60 breaths/min. Positive end expiratory pressure was set at 1 cm H_2_O. A polyethylene catheter was inserted into the left carotid artery to measure arterial pressure. A right heart catheter (PE 50 tubing) was inserted into the right ventricle through the right jugular vein for measurement of right ventricular systolic pressure with fluid-filled force transducers [10,11]. Cardiac output (CO) was measured by thermodilution technique as described [10,11]. Briefly, a thermistor (1.5 F) was placed into the ascending thoracic aorta via the right carotid artery for the measurement of transpulmonary thermodilution cardiac output (Cardiotherm 500-X, Hugo-Sachs Electronic – Harvard Apparatus GmbH, March-Hugstetten, Germany). The CO was averaged from three consecutive determinations and indexed to the weight of the animal to obtain cardiac index (CI). After exsanguination, the left lung was fixed for histology in 10% neutral buffered formalin and the right lung was snap frozen in liquid nitrogen.

### Right ventricular hypertrophy

The RV was dissected from the left ventricle (LV) and the septum (S) and weighed to determine the extent of RV hypertrophy as follows: RV/ (LV+S).

### Histological examination of the lungs

Paraffin lung sections (3 μm) were double-stained with anti-α-smooth muscle actin antibody (dilution 1:900, clone 1A4, Sigma, Saint Louis, Missouri) and anti-human von Willebrand factor antibody (vWF, dilution 1:900, Dako, Hamburg, Germany). Sections were counterstained with hematoxylin and examined by light microscopy using a computerized morphometric system (Qwin, Leica, and Wetzlar, Germany) for assessing the degree of muscularization of small peripheral pulmonary arteries [10,11,23]. In addition, lung sections were stained for Elastin-Nuclear Fast Red to assess the medial wall thickness.

Categorization of pulmonary arteries based on the degree of muscularization and on the external diameter and the percentage of medial wall thickness were performed as previously described [10,11,23].

### Microarray and data analysis

Total RNA extraction was performed using the Trizol reagent (Burlington, Ontario, Canada) according to the instructions given by the manufacturer from control, monocrotaline (MCT_[42d]_) and monocrotaline plus tolafentrine (MCT_[42d]_/Tola) treated lungs. Samples were treated with DNase I according to the RNase-free DNase set (QIAGEN, Hilden, Germany). Total RNA (5 μg) from each sample was used to generate Biotin-16-dUTP labeled cDNA and hybridized to the extracellular matrix and adhesion molecules gene array (GEArray Q series, Superarray, MD, USA). After hybridization, the membranes were developed according to the manufacturer's recommendations to yield luminescent signals, which were then captured on X-ray film.

The resulting image data were analyzed for differential gene expression patterns using GE Array Analyzer 1.2 (Super Array Bioscience Corp., Frederick, MD) software. Loading was adjusted on the basis of the intensity of hybridization signals relative to the housekeeping gene GAPDH.

### Real-time polymerase chain reaction

Quantitative real-time PCR was performed as described [26]. Briefly, total RNA was isolated from frozen lungs using Trizol Reagent according to the manufacturer's instructions. For the generation of cDNA, equal amounts of RNA from each sample were used as templates for reverse transcription of first-strand cDNA using the qPCR™ Mastermix (Euro-genetec, Seraing, Belgium) according to the manufacturer's protocol. For quantitative real-time RT-PCR analysis, 2 μl cDNA was placed into 50 μl reaction volume containing SYBR Green PCR mix and sequence-specific oligonucleotide primers. The thermal cycle conditions used for all reactions were as follows: activation, 50°C for 2 min; denaturation, 95°C for 10 min; and cycle, 95°C for 10 s and 60°C for 5 s (40 times). Specific primers used for sequence detection were: rGAPDH,5'-GTG ATG GGT GTG AAC CAC GAG-3' (forward) and5'-CCA CGA TGC CAA AGT TGT CA-3'(reverse); forMMP2,5'-ATC TGC AAG CAA GAC ATT GTC TT-3'(forward)and 5'-GCC AAA TAA ACC GAT CCT TGA A-3' (reverse);for MMP8, 5'-CGG GAA GAC ATA CTC TTC GAA-3'(forward)and 5'-CAT GGA TCT TCT TTG ATT GTC G-3' (reverse);for MMP9, 5'-GTA ACC CTG GTC ACC GGA CTT -3'(forward)and 5'-ATA CGT TCC CGG CTG ATC AG-3' (reverse); for MMP10, 5'- GGA GAT GCT CAC TTC GAT GAT-3' (forward)and 5'-CAG CAA CCA GGA ATA AAT TGG-3' (reverse); for MMP11, 5'-TTC TGA GAT TGA TGC TGC TTT C-3'(forward)and 5'-TGT CCA CGA AGG AAG TAG GC-3' (reverse); for MMP12, 5'-GCT GTC ACA ACA GTG GGA GA-3' (forward)and 5'-GTA ATG TTG GTG GCT GGA CTC-3' (reverse); for MMP20, 5'-GAA TAA ACT CTG GGG AAG CAG A-3'(forward)and 5'-TGA TTG GAT TAA GGC CTC GT-3' (reverse); for Icam, 5'-CAG CTG CGC TGT GTT TTG-3'(forward)and 5'-GGA TGG GAG CTG AAA AGT TG-3' (reverse); For Itgax, 5'-GCC TCG AGA CTG GAG ATC AT-3'(forward)and 5'-GGA GAG CTG GGA GCC AGT-3' (reverse);for Ncam1, 5'-ACC ATG AGC TGG ACA AAG GA-3'(forward)and 5'-CAC TGA AGA TGT GCT TCT CGT C-3' (reverse); for Plat, 5'-AAT GAA GGG AGA GCT GTT GTG-3'(forward) and 5'-TCC TCT TCT GAA CCT CCT GTG-3' (reverse); for Serpinb2, 5'-CGA AAG GGA TTT TGT GAT GTC-3' (forward)and 5'-GTG GGA AAT GGG AAT TCG T-3' (reverse). All real-time reactions were carried on an ABI 7700 sequence Detection System (Applied Biosystems, Foster City, CA, USA), and analysis was performed with the accompanying software. At the end of the PCR cycle, a dissociation curve was generated to ensure the amplification of a single product and the threshold cycle time (Ct values) for each gene was determined. Relative mRNA levels were calculated based on the Ct values and normalized to house keeping gene GAPDH.

### Migration assay

Pulmonary artery smooth muscle cell (PASMC) migration was examined in Transwell cell culture chambers with gelatin-coated polycarbonate membranes as described previously [27]. For this assay, control and MCT-treated rat PASMCs were isolated and added to the upper well of a Transwell (Corning Costar, Cambridge, MA) at 2 × 10^5 ^cells/well. The platelet-derived growth factor – BB (PDGF-BB) was added to the lower chamber at a concentration of 25 ng/ml and cells were allowed to migrate for 8 and 24 hours. In experiments with the combined selective PDE3/4 inhibitor tolafentrine (0.05, 0.1 and 0.3 μM), MCT-treated rat PASMCs were pre-incubated with tolafentrine in the upper well of a Transwell for 30 min before the addition of PDGF-BB into the lower chamber. Migration was quantified by staining the cells with crystal violet (Sigma, Deishofen, Germany) following microscopic cell counts on five random fields in each well. Experiments were performed in triplicate and were repeated at least three times.

### Data analysis

All data are given as mean ± SEM. Differences between the groups were assessed by analysis of variance (one-way ANOVA) and Student-Newman-Keuls test for multiple comparisons with a p value < 0.05 regarded to be significant.

## Results

### Acute vasodilatory effects of aerosolized tolafentrine in MCT treated (28d) rats

**Figure 1:**
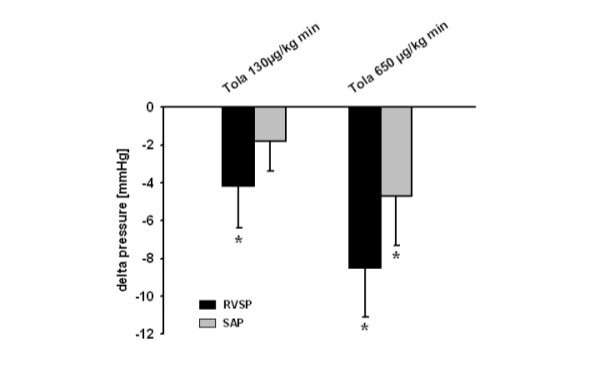
**Immediate vasodilatory effects of inhaled tolafentrine in monocrotaline-induced pulmonary arterial hypertension. **Monocrotaline (MCT_[28d]_) treated animals received tolafentrine over an inhalation period of 5 min subsequent to catheterization. The decrease in right ventricular systolic pressure (RVSP, in mmHg) and systemic arterial pressure (SAP, in mmHg) in response to the vasodilatory treatment is given. All values are given as mean ± SEM. *, p < 0.05 versus non treated animals

Aerosolized tolafentrine reduced right ventricular systolic pressure [RVSP] in MCT_[28d] _rats in a dose-dependent manner (Figure 1). As depicted, this pulmonary vasodilatation was accompanied by less pronounced decrease in systemic arterial pressure.

### Chronic effects of aerosolized tolafentrine: hemodynamics

**Figure 2:**
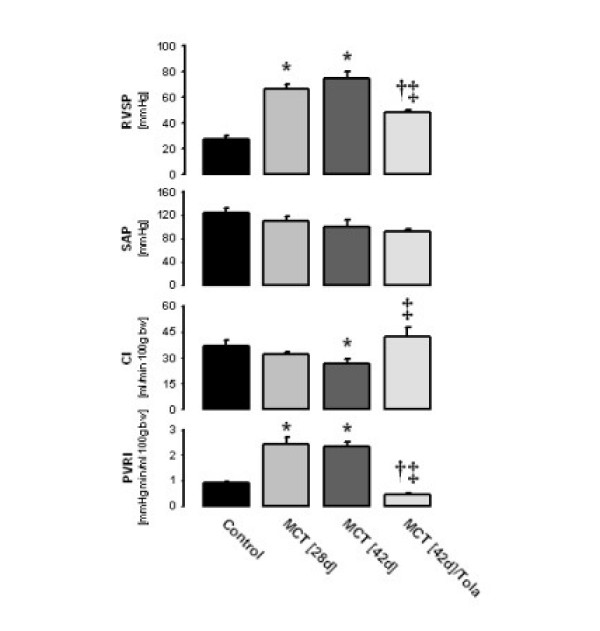
**Influence of inhaled tolafentrine on hemodynamics in monocrotaline – induced pulmonary arterial hypertension. **Right ventricular systolic pressure (RVSP, in mmHg), systemic arterial pressure (SAP, in mmHg), cardiac index (CI, in ml min^-1 ^100 g body weight^-1^) and total pulmonary resistance index (PRI, in mmHg min ml^-1 ^100 g body weight^-1^) are given. Tolafentrine was applied by repetitive inhalations from day 28 to day 42. All values are given as mean ± SEM. *, p < 0.05 versus control; †, p < 0.05 versus MCT_[28d]_; ‡, p < 0.05 versus MCT_[42d]_.

After injection of monocrotaline, pulmonary hypertension developed (right ventricular systolic pressure on day 28 = 66.5 ± 3.2 mm Hg (n = 11) and on day 42 = 74.9 ± 5.1 mm Hg (n = 9), as compared to 25.9 ± 4.0 mm Hg in the control animals (n = 10)) (Figure 2). No significant changes in systemic arterial pressure occurred. As compared to control animals (36.5 ± 3.5 ml/min 100 g body weight), cardiac index was slightly decreased on day 28 (31.8 ± 1.3 ml/min/100 g body weight) and significantly decreased on day 42 (28.1 ± 2.5 ml/min/100 g body weight). Aerosolized tolafentrine treatment significantly lowered right ventricular pressure to 48.4 ± 2.1 mmHg (p < 0.05 versus MCT_[42d] _and MCT_[28d]_). No significant changes in systemic arterial pressure occurred in animals undergoing long-term tolafentrine inhalation (n = 8), while cardiac output was significantly increased in the MCT_[42d]_/Tola group.(42.1 ± 5.1 ml/min/100 g body weight; p < 0.05 versus MCT_[42d]_). The total pulmonary resistance index, calculated from the RVSP and cardiac output values, was even fully normalized in the MCT_[42d]_/Tola animals.

### Chronic effects of aerosolized tolafentrine: right ventricular hypertrophy

**Figure 3:**
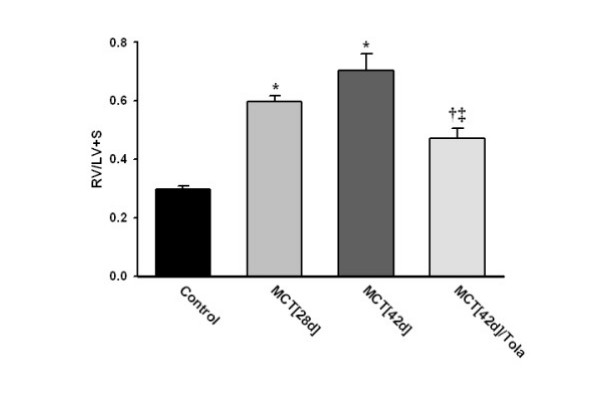
**Influence of inhaled tolafentrine on right heart hypertrophy. **Right to left ventricular plus septum ratio (RV/LV+S) of different treatment groups is given. Tolafentrine was applied by repetitive inhalations from day 28 to day 42. All values are given as mean ± SEM. *, p < 0.05 versus control; †, p < 0.05 versus MCT_[28d]_; ‡, p < 0.05 versus MCT_[42d]_.

Four weeks after injection of MCT, animals demonstrated significant right heart hypertrophy, as indicated by an increase in the right ventricular to left ventricular plus septum weight ratio (RV/LV+S) from 0.29 ± 0.02 (control animals) to 0.60 ± 0.02 (Figure 3). Rats that received inhaled vehicle for 2 weeks demonstrated further progression of right ventricular hypertrophy (RV/LV+S = 0.71 ± 0.05). Inhaled tolafentrine reversed established right ventricular hypertrophy (MCT_[42d]_/Tola = 0.47 ± 0.03; p < 0.05 versus MCT_[28d] _and MCT_[42d]_) (Figure 3).

### Chronic effects of aerosolized tolafentrine: survival

In this study, rats were randomized to receive vehicle or inhaled tolafentrine beginning four weeks after MCT treatment. In the MCT_[28d] _group a survival of 78% was noted, while survival decreased to 60% in the MCT_[42d] _group. In the MCT_[42d]_/Tola 80 % survived the treatment protocol. However, due to insufficient number of experiments the significant effects on this parameter were not shown in detail.

### Chronic effects of aerosolized tolafentrine: histopathology

**Figure 4:**
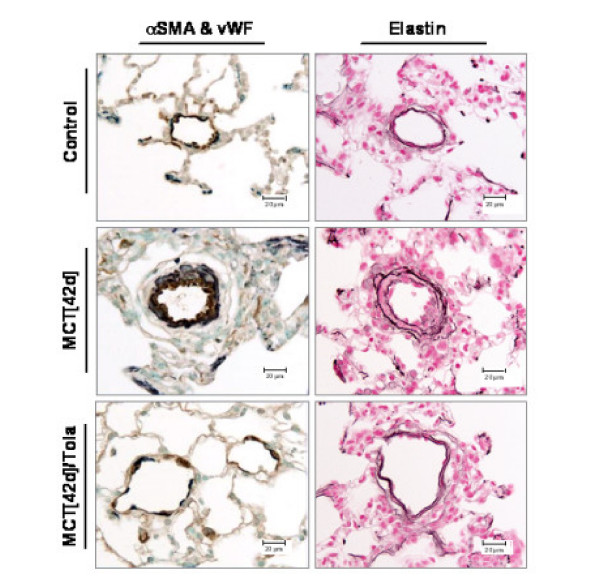
**Effect of inhaled tolafentrine on the degree of muscularization and on the medial wall thickness of small pulmonary arteries. **Immunohistochemical analysis of lung sections originating from saline (Control), monocrotaline (MCT_[42d]_) and monocrotaline plus tolafentrine (MCT_[42d]_/Tola) treated animals. Staining was undertaken for von Willebrand-factor (brown; endothelial cells) and alpha smooth muscle actin (purple; smooth muscel cells) as well as elastin. Scale bar: 20 μm.

**Figure 5:**
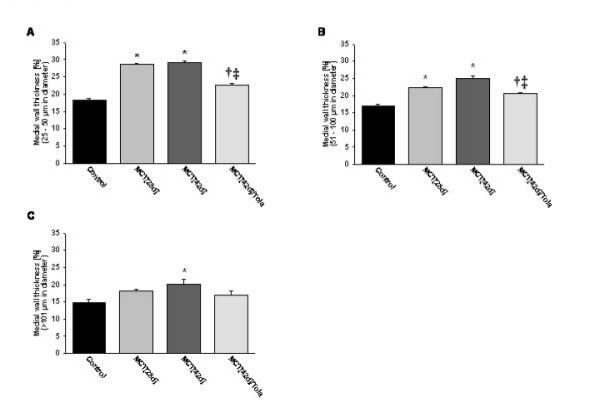
**Effect of inhaled tolafentrine on medial wall thickness of pulmonary arteries. **Measurement of medial wall thickness (given in percentage of total wall thickness) of pulmonary arteries sized from (A) 25 to 50 μm, (B) 51–100 μm and (C) >101 μm. All values are given as mean ± SEM. *, p < 0.05 versus control; †, p < 0.05 versus MCT_[28d]_; ‡, p < 0.05 versus MCT_[42d]_.

**Figure 6:**
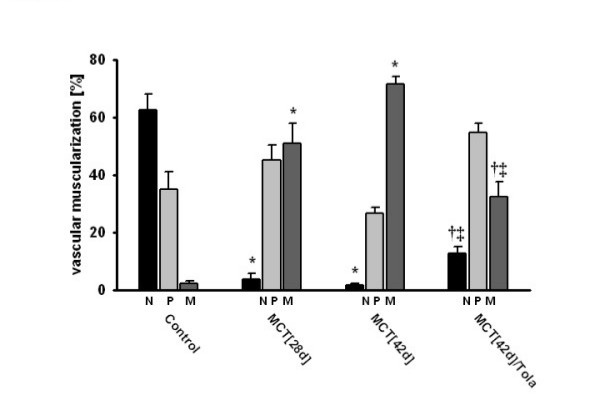
**Effect of inhaled tolafentrine on the degree of muscularization of peripheral pulmonary arteries. **Percentage of non- (N), partially (P) or fully (M) muscularized pulmonary arteries, related to the total number of pulmonary arteries is given. A total of 60 to 80 intra-acinar vessels were analysed in each single lung from saline (Control), monocrotaline (MCT_[28d] _and MCT_[42d]_), and monocrotaline plus tolafentrine (MCT_[42d]_/Tola) treated animals. Tolafentrine was applied by repetitive inhalations from day 28 to day 42. All values are given as mean ± SEM. *, p < 0.05 versus control; †, p < 0.05 versus MCT_[28d]_; ‡, p < 0.05 versus MCT_[42d]_.

Elastin staining and subsequent morphometric analysis of pulmonary arteries demonstrated a markedly increased medial wall thickness in both the MCT_[28d] _and the MCT_[42d] _groups, when compared with the saline-treated group (Figure 4). In comparison to control animals, the percentage of medial wall thickness of arteries sized between external diameters of 25 to 50 μm was increased significantly from 18.3 ± 0.42 to 28.5 ± 0.33 (MCT_[28d]_) and 29.1 ± 0.48 (MCT_[42d]_) (Figure 5A). To a similar extent, the percentage of medial wall thickness of arteries with external diameters of 51–100 μm increased from 17 ± 0.49 (control) to 22.2 ± 0.31 (MCT_[28d]_) and 25.1 ± 0.77 (MCT_[42d]_) (Figure 5B). In arteries larger than 100 μm, the corresponding data were 14.8 ± 0.95 (control), 18.1 ± 0.57 (MCT_[28d]_) and 20.6 ± 1.44 (MCT_[42d]_) (Figure 5C), respectively. The evaluation of the extent of muscularization of pulmonary arteries with external diameters of 15 to 50 μm demonstrated a significant reduction in non-muscularized pulmonary arteries (62.6 ± 5.5% in controls, 3.7 ± 2.1 % in MCT_[28d]_, 1.6 ± 0.7 % in MCT_[42d]_). Concomitantly, a significant increase in fully muscularized vessels from 2.4 ± 0.62 (control) to 51.1 ± 6.85 (MCT_[28d]_) and 71.6 ± 2.63 (MCT_[42d]_) was noted (Figure 6).

Most impressively, both, medial wall thickness (22.5 ± 0.65%) and percentage of fully muscularized pulmonary arteries (32.5 ± 5) were significantly reduced by long term treatment of aerosolized tolafentrine, while the percentage of non-muscularized pulmonary arteries significantly increased.

### Comparison of migratory responses of control- and MCT-PASMCs

**Figure 7:**
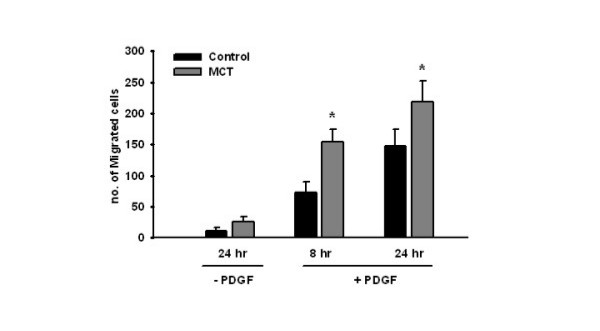
**Comparison of migratory responses of control and MCT – PASMCs. **Migratory responses of control and monocrotaline rat PASMCs were compared at different time points in presence of PDGF-BB (25 ng/ml). The number of migrated cells was counted in 5 random areas per membrane. The results are based on three different independent experiments. All values are given as mean ± SEM. *, p < 0.05 versus control PASMCs at the respective time points.

Migration of control and MCT-treated rat PASMCs was examined at 8 and 24 h in the presence of the chemo-attractant PDGF-BB (Figure 7). In the presence of PDGF-BB, the migration rate of PASMCs derived from MCT rats ranged at 207% and 155% of that of the PASMCs derived from control rats (8 hr and 24 hr data, respectively).

### Effect of tolafentrine: PDGF-Induced MCT- PASMCs migration

**Figure 8:**
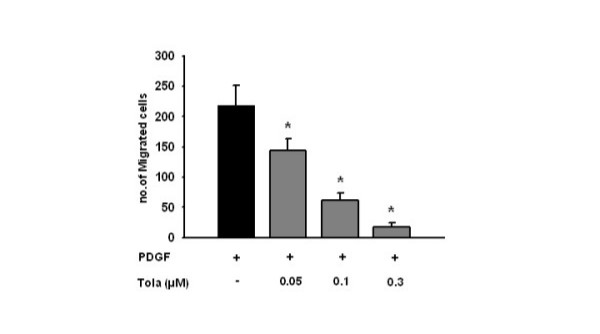
**Effect of tolafentrine on PDGF-directed PASMCs migration. **Tolafentrine at different concentrations (0.05, 0.1, and 0.3 μM) inhibited PDGF-BB (25 ng/ml)-induced MCT-treated rat PASMCs migration. The number of migrated cells was counted in 5 random areas per membrane. The results are based on three independent experiments. All values are given as mean ± SEM. *, p < 0.05 versus PDGF-BB stimulated PASMCs.

Inhibitory effects of tolafentrine on MCT- treated rat PASMCs migration were examined using a migration assay with modified Boyden chamber. The PDGF caused strong enhancement of PASMC migration. Treatment with tolafentrine inhibited PDGF induced PASMC migration in a dose-dependent fashion: at 0.05, 0.1, and 0.3 μM tolafentrine, PASMC migration was reduced by 34 %, 72 %, and 92 % (Figure 8).

### Effects of tolafentrine: expression of matrix-degrading proteases

**Figure 9:**
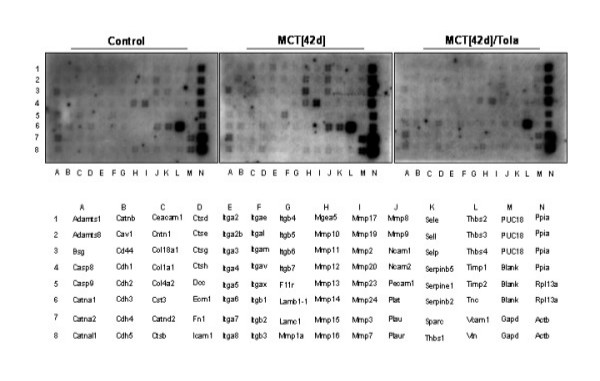
**Effect of tolafentrine on MMP and adhesion molecule gene expression – cDNA micro-array analysis. **Biotin labeled cDNA probes generated from total RNA from control lungs (Control), monocrotaline (MCT_[42d]_), and monocrotaline plus tolafentrine (MCT_[42d]_/Tola) treated lungs were hybridized to the identical extracellular matrix and adhesion molecules gene arrays. Hybridization patterns were assessed by chemiluminescence. Arrays representative of three independent array experiments per group are shown.

To study the mechanisms underlying tolafentrine-inhibited PASMC migration, lung samples from control, MCT_[42d] _and MCT_[42d]_/Tola rats were analyzed on 96 gene arrays encoding key extracellular matrix and adhesion molecules (representative arrays given in Figure 9). The analysis shows expression of 12 of the 96 extracellular matrix and adhesion related genes in the control lungs. Further, a total of 12 genes were differentially expressed and modulated in the MCT-treated lungs. The up-regulated genes include 7 MMPs (MMP 2, MMP 8, MMP 9, MMP 10, MMP 11, MMP 12, MMP 20), 1 serine protease (Plat), 3 cell adhesion molecules (ICAM-1, NCAM-1, Itgax), and 1 protease inhibitor (Serpinb2).

**Figure 10:**
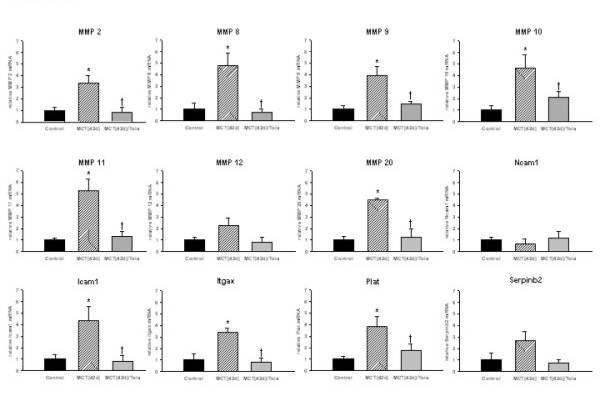
**Quantitative real-time RTPCR confirmation of relative changes detected in micro arrays. **Relative quantification of mRNAs encoding for MMP 2, MMP 8, MMP 9, MMP 10, MMP 11, MMP 12, MMP 20, Icam, Itgax, Ncam, Plat, and Serpinb2 related to the housekeeping gene GAPDH was undertaken by real-time RTPCR. The lung samples originated from control, monocrotaline (MCT_[42d]_), and monocrotaline plus tolafentrine (MCT_[42d]_/Tola) treated animals. All values are given as mean ± SEM. *, p < 0.05 versus control, †, p < 0.05 versus MCT_[42d]_.

To validate these findings, the expression of 12 genes was investigated by quantitative real-time PCR. These results confirmed the array data for most of the proteases investigated (Figure 10). Monocrotaline treatment for four weeks caused an upregulation of the gelatinases MMP 2 and MMP 9 by a factor of 3.39 ± 0.65 and 3.92 ± 0.77, respectively. MMP 8, the interstitial collagenase, was elevated 4.77 ± 1.44 fold with MCT treatment. In addition, the stromelysins MMP 10, MMP 11, MMP 12 and MMP 20 were significantly upregulated by factors of 4.65 ± 1.13, 5.27 ± 1.03, 2.27 ± 0.63 and 6.67 ± 0.75, respectively.

Several adhesion molecules were also differentially expressed at the mRNA level. We observed a significantly increased expression of Icam and Itgax (4.34 ± 1.22 and 3.44 ± 0.33 fold). In contrast, Ncam was not changed upon MCT treatment. An upregulation of tissue plasminogen activator (plat) by 3.74 ± 0.83 and plasminogen activator inhibitor-2 (Serpinb2) by 2.68 ± 0.83 foldwith MCT treatment was also observed.

Most impressively, virtually all alterations in extracellular matrix and adhesion molecule gene expression induced by monocrotaline were fully or partially abrogated by tolafentrine (12 genes given in Figure 10).

## Discussion

In the present study, we demonstrate that daily repetitive tolafentrine inhalation significantly improved pulmonary hemodynamics and reversed structural and molecular changes underlying MCT induced PAH in rats. Notably, the inhalative therapy was commenced after full establishment of pulmonary hypertension, 4 weeks after application of monocrotaline. Similar to the abnormalities in human PAH, monocrotaline treatment in rats is known to provoke endothelial injury, proliferation, migration and hypercontraction of vascular smooth muscle cells, as well as inflammatory sequelae [28,29]. The animals die due to a progressive increase in precapillary lung vascular resistance with subsequent right heart failure.

A recent study by our group showed that intravenous infusion of the combined selective PDE 3/4 inhibitor (tolafentrine) prevented the development of pulmonary hypertension and right ventricular hypertrophy in response to monocrotaline [11]. However, the complexity and complications associated with the intravenous application of an agent exerting at the same time pulmonary and systemic vasodilation prompted us to evaluate the inhalative route of application in the present study. Moreover, the *therapeutic *potential of tolafentrine, i.e. its efficacy after full establishment of severe pulmonary hypertension, and its impact on molecular mechanisms closely linked with the structural wall changes were not addressed in the previous study.

For the inhalation therapy, aimed to achieve a selective pulmonary vasodilation, a 15 fold lower dose compared with the intravenous route of application was employed (120 μg/kg day versus 2 mg/kg day). This low inhaled dosage exerted per se no acute effects on systemic hemodynamics but demonstrated selective pulmonary vasodilation as has been demonstrated for several other compounds [16,17]. Further increase in dose results in spill over of the compound in the systemic circulation and systemic side effects as demonstrated by the higher dose of 650 μg tolafentrine/kg min in this study. Since PDE 3 and 4 are expressed in smooth muscle cells throughout the cardiovascular system, tolafentrine was nebulized in a dose which cause some direct lung vasorelaxation but which is still too low to provoke systemic vasodilatory effects. However, the results of an acute test of inhaled tolafentrine under general anesthesia may not reproduce the effects in an awake animal, but long term nebulization demonstrated strong anti-remodeling effects in the setting of MCT-induced PH.

Nevertheless, and notwithstanding the late initiation of the tolafentrine treatment, hemodynamics were dramatically improved after 2 weeks of inhalative tolafentrine treatment: RVSP values were markedly lower than those before onset of treatment, and cardiac index as well as total pulmonary resistance index were also fully normalized. Accordingly, the right heart hypertrophy was found to be largely decreased, as were the structural changes of the lung vasculature evoked by monocrotaline treatment. The increase in the medial thickness of the precapillary lung arteries was reversed, and the high percentage of fully muscularized peripheral pulmonary arteries decreased in response to tolafentrine inhalation. To our knowledge, this is the first time that combined selective PDE 3/4 inhibition (tolafentrine) has been shown to reverse established pulmonary hypertension both with respect to hemodynamics and the structural remodeling of the lung vasculature.

These findings suggest a potent anti-proliferative effect of combined selective PDE 3/4 inhibition in the lung vasculature, as has been suggested by preceding in vitro data. Selective and nonselective cAMP PDE inhibitors have been shown to elicit a concentration-dependent attenuation of mitogen-induced proliferation in rat and human PASMC predominantly via adenylyl cyclase and protein kinase A [30,31]. Furthermore, PASMC receiving the combination of a PDE3 and a PDE4 inhibitor exhibited a significant additive or synergistic anti-mitogenic effect as compared to each PDE inhibitor used on its own.

Interestingly, an enhanced migratory capacity in response to PDGF was observed in PASMCs isolated form lungs of MCT treated rats as compared to matched control lungs. This is reminiscent of the reported higher migratory response of aortic SMCs derived from spontaneously hypertensive rats as compared to normotensive rats [31,32]. Although the mechanisms responsible for the differential migratory responses of SMC are not known, increased collagenase activities might partly explain this phenomenon, since the destruction of extracellular matrix barriers is an important early step for SMC migration [33,34]. Moreover, enhanced MAP kinase activities, such as extracellular signal-regulated kinase (ERK) and p38 kinase [35], might play a role in PDGF-induced vascular SMC migration, which is in line with the observation of increased ERK expression in the precapillary arteries of MCT-treated rats (data not shown).

The present study demonstrates for the first time that selective PDE 3/4 inhibition inhibits the migration of SMCs stimulated with PDGF in a concentration-dependent manner. The PDGF-BB-induced SMC migration was significantly inhibited by 0.05–0.3 μM tolafentrine. This is well in line with the study of T. Horio et al, who reported that adrenomedullin inhibits the migration of aortic SMCs in response to PDGF, probably through a cAMP-dependent process [36]. Downstream effects of elevated cAMP levels, being relevant for the SMC migratory response, may include MAP kinase signaling and cytosolic Ca^2+ ^regulation [37,38].

**Figure 11:**
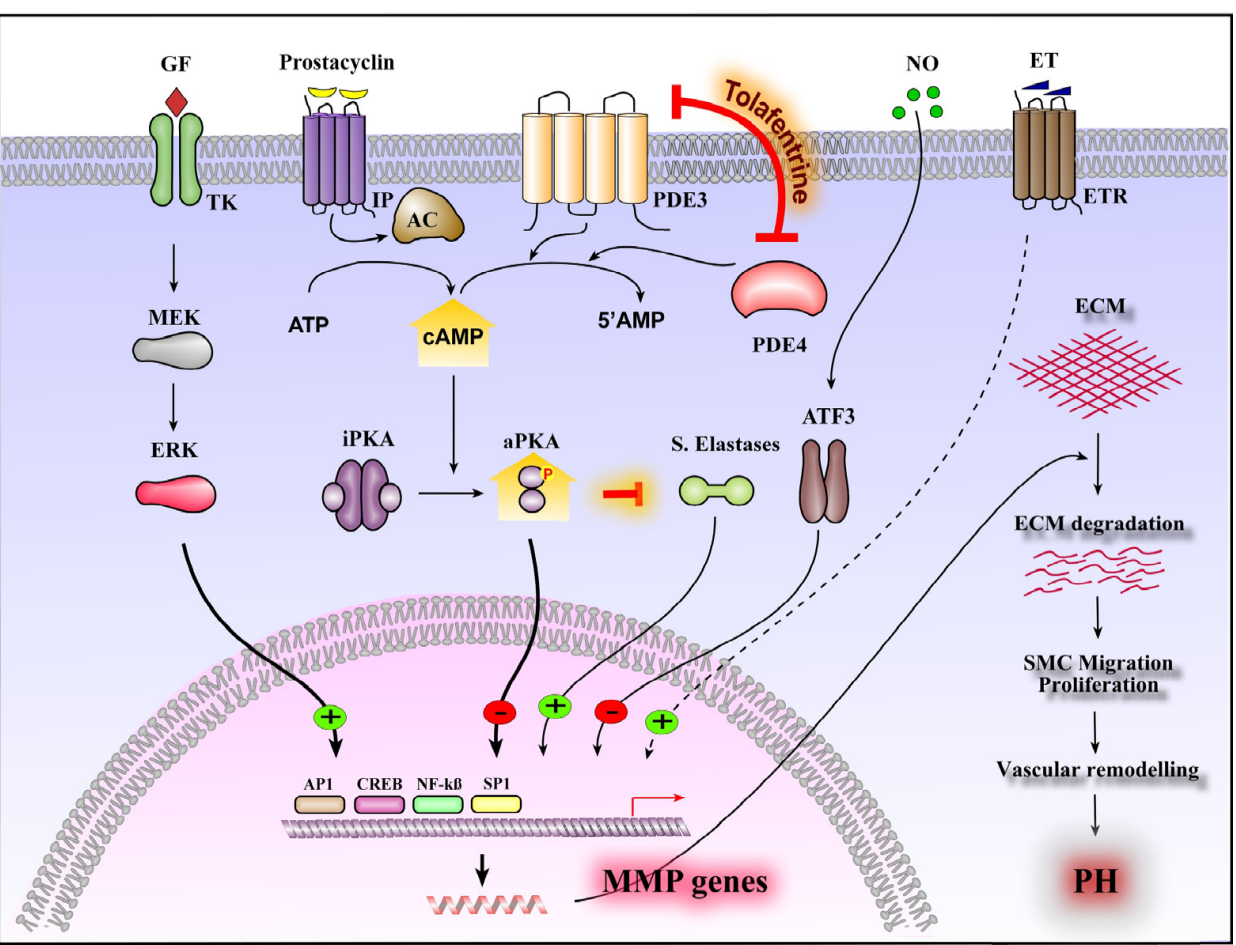
**Potential interactions among various mediator pathways in the regulation of MMP expression and in the subsequent development of PH. **Schematic depiction of molecular mechanisms responsible for MMP transcriptional regulation. Growth factors such as tyrosine kinases (TK), endothelin system (ET) and serine elastases (S.Elastases) positively modulate several MMP genes transcription. MMPs that were produced then cleave extracellular matrix (ECM) and thereby promotes SMC migration and proliferation. Augmentation of these processes ultimately leads to pulmonary vascular remodeling and PH. In contrast, mediators that influence cAMP pathway (Prostacyclin analogues (PGI_2_) and combined PDE 3/4 inhibitors (tolafentrine) and to a less extent nitric oxide (NO) represses MMP gene activation that were positively modulated by various growth factors during development of PH. These effects eventually lead to regression of matrix degradation, SMC migration and proliferation and reverse-remodeling of pulmonary arteries. GF, growth factor; TK, tyrosine kinase; MEK, MAP/ERK kinase; ERK, Extracellular signal-regulated kinase; IP, prostaglandin I2 (prostacyclin) receptor; AC, adenylyl cyclase; PDE3, phosphodiesterase isoenzyme 3; PDE4, phosphodiesterase isoenzyme 4; iPKA, inhibitory protein kinase A; aPKA, activated protein kinase A; S.Elastases, serine elastases; NO, nitric oxide; ATF3, activating transcription factor 3; ET, endothelin; ETR, endothelin receptor; AP1, activator protein 1; CREB, CRE-binding protein; NFkβ, Sp1, Sp1, transcription factor; NF-kB, nuclear factor-kappaB (p65); MMP, matrix metallo proteases; ECM, extracellular matrix.

The anti-proliferative and anti-migratory effects of tolafentrine may be closely linked to matrix regulation. Our group and others have previously demonstrated an increased expression of gelatinases, MMP 2 and MMP 9 in monocrotaline induced pulmonary hypertension [5,11]. In order to approach this field in more depth, gene array analysis of proteinases and adhesion molecules and subsequent confirmation with real time PCR was undertaken in the present study. A strong upregulation of various extracellular matrix and adhesion molecule genes in response to monocrotaline was noted, in particular MMPs (MMP 2, 8, 9, 10, 11, 12, 20), serine proteases (Plat and Serpinb2) and cell adhesion molecules (Icam, Ncam and Itgax). These observations strongly support the notion that increased MMP expression and activity directly correlates with the severity of disease in various experimental forms of pulmonary hypertension. Several mechanisms may be responsible for MMP upregulation in pulmonary arteries during PAH (Figure 11). The MMP expression may be stimulated by growth factors (PDGF, EGF), cytokines, most notably interleukin IL-1α, and physical forces, which may be induced after MCT administration. Several of these factors stimulate a cascade of MAP kinases and PKC and potently activate the transcription factors AP-1 and NF-kB, which are involved in MMP and adhesion molecule expression [39,40]. Most notably, tolafentrine treatment resulted in strong down-regulation or even normalization of the transcription of most of the MMP and adhesion molecule genes that were upregulated in response to MCT. Moreover, the inhibitory effect of tolafentrine on MMPs was functionally confirmed, since it significantly reduced the migratory properties of PASMCs in vitro. This most impressive regression of MMP and adhesion molecule expression under tolafentrine may in part be explained by cAMP-mediated regulation of cytokines and growth factors, being upstream effectors of MMPs (Figure 11). In addition, cAMP has been shown to inhibit the NF-kB transcriptional activity via protein kinase A [41-43]. Such impact might also explain the down-regulation of Icam and Ncam by tolafentrine, as these adhesion molecules are also known to be NF-kB dependent [44].

## Conclusion

In conclusion, we demonstrate for the first time that inhalation of a combined selective PDE3/4 inhibitor reverses pulmonary hypertension fully developed in response to monocrotaline in rats. This "reverse-remodeling" effect was true for hemodynamics, structural changes to the lung vascular wall, and key molecular pathways of matrix regulation. We provide evidence that inhibition of pulmonary artery SMC migration and MMP-based matrix regulation play a major role in the beneficial effect of inhaled tolafentrine in severe PAH. Reversal of structural lung vascular remodeling may apparently be achieved in pulmonary hypertension, at least in the experimental model of monocrotaline-induced PAH in rats.
